# First Outbreak of Aeromoniasis, Caused by *Aeromonas veronii,* in Farmed European Seabass (*Dicentrarchus labrax*) in the Ionian Sea, Greece

**DOI:** 10.3390/pathogens14060587

**Published:** 2025-06-14

**Authors:** Eirini Lampou, Eleni Psychari, Konstantina Louka, Charalampos Kotzamanidis, Andigoni Malousi, Ioannis Petropoulos, Markos N. Kolygas, Dimitrios Doukas, Konstantina Bitchava

**Affiliations:** 1 Department of Aquaculture and Fish Health, Pharmaqua S.A., Metamorfosi, 14451 Athens, Greece; e.lampou@pharmaqua.gr (E.L.); e.psychari@pharmaqua.gr (E.P.); konstantinaloukas@gmail.com (K.L.); 2 Department of Aquaculture and Fish Diseases, Faculty of Veterinary Science, University of Thessaly, 43100 Karditsa, Greece; kolygasmarkos@gmail.com; 3 Veterinary Research Institute, Hellenic Agricultural Organization DIMITRA, 57001 Thessaloniki, Greece; kotzam@elgo.gr; 4 Laboratory of Biological Chemistry, Medical School, Aristotle University, 54124 Thessaloniki, Greece; andigoni@auth.gr; 5 Laboratory of Applied Hydrobiology, Department of Animal Science, School of Animal Biosciences, Agricultural University of Athens, 11855 Athens, Greece; yiannis_efpalio@yahoo.gr; 6 Laboratory of Animal Pathology & Veterinary Forensics, Faculty of Veterinary Science, University of Thessaly, 43100 Karditsa, Greece; ddoukas@uth.gr

**Keywords:** seabass, *Dicentrarchus labrax*, *Aeromonas veronii*, aeromoniasis, outbreak, Ionian Sea

## Abstract

This article documents the first outbreak of aeromoniasis caused by *Aeromonas veronii* in farmed European seabass (*Dicentrarchus labrax*) along the Greek Ionian Sea coast. In late spring 2024, commercially sized fish exhibited anorexia, hemorrhages, and ulcers on the skin, accompanied by elevated morbidity and mortality rates. The outbreak spread rapidly across local farms in Sagiada Bay, reaching its peak in late summer, and extending into the Astakos Gulf, southern in the Ionian Sea. The postmortem examination revealed hemorrhages, organomegaly, abscess formation, and granulomatous inflammation. *Aeromonas veronii* was isolated from all examined individuals in nutrient media and confirmed by biochemical and molecular methods. Whole genome sequencing and phylogenetic analysis demonstrated genetic homogeneity among two strains from two different areas along the Ionian Sea and a close evolutionary relationship with other *Aeromonas veronii* strains from the Aegean Sea. Although genetically similar, the isolates exhibited differences in phenotypic and biochemical characteristics, indicating regional variability. The present study provides an overview of the pathology, clinical characteristics and progression of aeromoniasis in Ionian Sea aquaculture, highlighting the need for continued monitoring, in-depth genomic and phenotypic assessment, and the design of region-specific preventive strategies, including autogenous vaccines, for effective disease management.

## 1. Introduction

Aquaculture has developed into a crucial global industry, significantly contributing to the rising demand for seafood. Among the diverse species cultivated within this industry, Mediterranean favorites like European seabass (*Dicentrarchus labrax*) and Gilthead seabream (*Sparus aurata*) are especially valued for their distinctive flavors and substantial marketability.

In recent years, Mediterranean aquaculture has emerged as a significant contributor to global fish production. In 2023, the overall production of Mediterranean farmed fish species reached an impressive 615,000 tons, with a total value of EUR 697 [1]. Notably, Greece has established itself as a powerhouse in this sector, contributing about 58% of the EU’s seabream and seabass production and 20% of the international market [2].

However, as aquaculture production scales up to meet rising consumer demand, it faces numerous challenges. Disease outbreaks in farmed fish pose significant threats to production, impacting both economic viability and food security [3]. Particularly bacterial infections are among the most detrimental factors affecting aquaculture systems, leading to considerable morbidity and mortality rates [4].

One of the important pathogens is *Aeromonas veronii*, a gram-negative bacterium frequently associated with aquatic habitats [5]. This organism is known for its capacity to induce systemic infections in fish, leading to severe losses in aquaculture species. These include: channel catfish (*Ictalurus punctatus*) [6], cyprinid fish [7], Chinese sucker (*Myxocyprinus asiaticus*) [8], freshwater sleeper (*Odontobutis potamophila*) [9], crucian carp (*Carassius auratus gibelio*) [10], Nile tilapia (*Oreochromis niloticus*) [11,12], rohu (*Labeo rohita*) [13], and largemouth bass (*Micropterus salmoides*) [14]. Furthermore, *Aeromonas veronii* has recently been isolated from wild fish after it was associated with an outbreak causing significant disease in bronze gudgeon (*Coreius heterodon*) [15]. In Mediterranean fish species, this bacterium has been associated with severe losses in seabass production both in the Aegean Sea and the Black Sea [16,17,18].

This paper presents the first recorded outbreak of disease caused by *Aeromonas veronii* in seabass along the Greek coasts of the Ionian Sea, highlighting the clinical manifestations, the diagnostic approach and providing a comprehensive genomic characterization of the isolated strains. Through this investigation, we aim to contribute to the broader understanding of *Aeromonas veronii* as a significant threat to fish populations, thereby paving the way for improved disease management strategies in the aquaculture industry.

## 2. Materials and Methods

### 2.1. Disease Description

**Figure 1:**
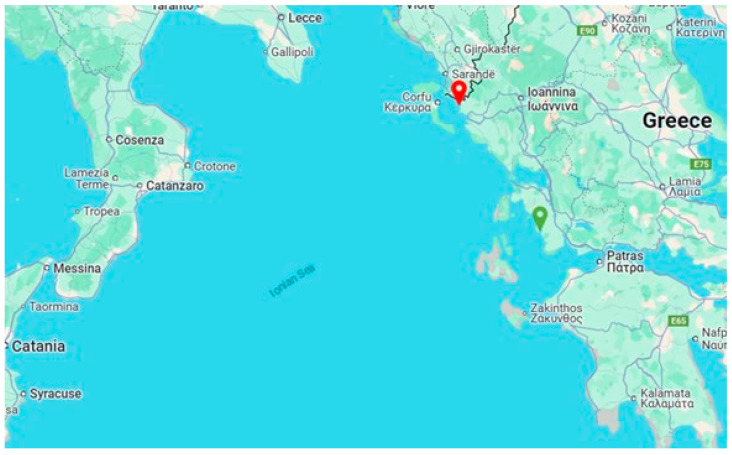
A map of the Ionian Sea, located between southern Italy and western Greece, with key locations marked. The red location symbol indicates Sagiada Bay, while the green symbol denotes the Astakos Gulf (Source: Google Maps).

The initial clinical manifestations of the disease were documented in a fish farm located in Sagiada Bay, Thesprotia, Greece, during late Spring 2024, when the water temperature was recorded at 18 °C. By June, the disease had spread to nearby fish farms, reaching its peak incidence in July and August, at water temperatures ranging from 26 °C to 29 °C. By late summer, the disease had emerged in the Astakos Gulf, which lies in the southern part of the Ionian Sea (Figure 1).

The affected fish, all weighing over 250 g, exhibited anorexia and lethargic swimming at the water surface, followed by abrupt diving to the bottom. Notable external signs included paleness of the dorsal surface of the skin, pale to yellow color and petechiae on the ventrum and the bases of the fins, and hemorrhages and distension of the cloaca. Occasionally, orbital erosions and ulcers on the skin were observed, as well as ocular lesions such as corneal ulcers and exophthalmos.

Daily mortality rates varied from 0.01% to 0.05% based on water temperature and stocking density. The use of antibiotics, based on antimicrobial susceptibility tests, resulted in a short-term decrease in mortality, but losses returned to initial levels after approximately 20 days. Consequently, several treatment cycles were implemented in many cases. The losses persisted until the end of autumn, resulting in cumulative mortalities between 15% and 20% in different farms.

### 2.2. Postmortem Examination

Moribund seabass, exhibiting distinct clinical signs of disease, were collected for postmortem examinations. Following the initial identification of the disease, additional samples were systematically collected from various nearby fish farms to monitor pathogen dissemination. In total, 524 individuals from seven fish farms were examined. Upon arrival, necropsies were performed to assess the health status of each individual, and tissue samples were obtained for parasitological, microbiological, molecular, and histological analyses.

### 2.3. Histology

Samples from liver, kidney, and spleen, collected during necropsy, were fixed in 10% neutral buffered formalin and routinely embedded in paraffin wax. The sliced sections (5 µm) were stained with hematoxylin and eosin (H&E) [19] and Ziehl–Neelsen (ZN) and observed under a light microscope (microscope: Olympus CX23 and camera: Olympus EP50 by Olympus, Tokyo, Japan).

### 2.4. Microbiology

Bacteria were isolated by streaking head kidney tissue from of affected fish onto blood agar (BA), Thiosulfate-citrate-bile salts-sucrose (TCBS) agar, Trypticase soy agar (TSA) supplemented with 2% NaCl and Mueller–Hinton agar (MH) (Bioprepare Microbiology, Athens, Greece) according to the methods described by R. J. Roberts [20]. The plates were incubated at 25 °C for a total duration of 72 h. Colony morphology was evaluated on all types of agars, while brown pigment production and hemolytic activity were assessed on MH and BA, accordingly. The observations were recorded at 18, 24, 48, and 72 h.

The morphology of the bacteria was evaluated after microscopic examination of wet mounts and Gram-stained smears under light microscope (microscope: Olympus CX23, camera: Olympus EP50).

Antimicrobial susceptibility testing was determined by the disk diffusion method using commercially available disks (Oxoid Ltd., Basingstoke, UK) [21]. The suspension of a single colony was inoculated onto Mueller–Hinton agar, and inhibition zones were measured after incubation at 25 °C for 24 h. The antimicrobial agents tested were: oxolinic acid (OA, 2 µg), florfenicol (FFC, 30 µg), oxytetracycline (OT, 30 µg), trimethoprim/sulfamethoxazole (SXT, 25 µg), doxycycline (DO, 30 µg), and flumequine (UB, 30 µg), the ones approved in Greece for use in aquaculture. The results were classified as resistant (R), intermediately resistant (I), or susceptible (S) according to the CLSI guidelines [22,23].

### 2.5. Biochemical Testing

From the total of the isolates, four (Thesp1, Thesp2, Thesp3, Aetol1) were subjected to biochemical analysis using the GNA+B-ID System (Microgen Bioproducts Ltd., Camberley, UK) and VITEK^®^ 2 Automated System (Biomerieux, Marcy-l’Étoile, France), adhering to the manufacturer’s guidelines. Motility tests (Bioprepare Microbiology, Athens, Greece), oxidase tests (Biomerieux, Marcy-l’Étoile, France), and catalase tests (Liofilchem S.r.l, Roseto degli Abruzzi, Italy) were carried out separately.

### 2.6. Biofilm Formation Ability of Isolates

The biofilm-forming capability of *Aeromonas veronii strains* was evaluated through a semi-quantitative microplate assay, employing a crystal violet staining technique to measure the optical density (OD) of the adhered biofilms.

In brief, *Aeromonas veronii* isolates were cultured overnight at 37 °C in TSB and then diluted to 108 CFU/mL using the same medium. Two hundred microliters of each culture were meticulously transferred into individual wells of a 96-well polystyrene microtiter plate (Cole-Parmer, Vernon Hills, IL, USA) and incubated aerobically at 37 °C for 24 h. Subsequently, each well was washed three times with 200 μL of sterile 0.9% NaCl to remove loosely adhering cells. The samples were then stained by adding 100 μL of a 0.3% (*w*/*v*) crystal violet solution. The excess staining was removed by carefully rinsing with water three times. After destaining with ethanol, the microtiter plate was allowed to air dry, and the optical density of the adhered biofilms was measured spectrophotometrically at a wavelength of 570 nm. The cut-off optical density (ODc) was established as three standard deviations above the average optical density of the negative control. Based on the resulting OD readings, *A. veronii* strains were categorized following the criteria established by Borges et al. [24] as no biofilm producers (OD < ODc), weak biofilm producers (ODc < OD ≤ 2 × ODc), moderate biofilm producers (2 × ODc < OD ≤ 4 × ODC), or strong biofilm producers (4 × ODc < OD). All tests were conducted three times, and the results from the microtiter plates were averaged for accuracy.

### 2.7. DNA Extraction and Real-Time PCR

A total of 23 pooled spleen tissue samples and 21 bacterial colonies were examined. Total DNA was extracted from 25 mg of pooled spleen tissue and bacterial colonies, isolated from the kidneys of moribund individuals, using the Genesig Easy DNA/RNA Extraction Kit (Primerdesign Ltd., Chandler’s Ford Eastleigh, UK) according to the manufacturer’s instructions.

The DNA extracts from the colonies were subjected to real-time PCR for the detection of *Aeromonas veronii*, while the spleen samples were analyzed for the presence of *Aeromonas veronii*, *Photobacterium damselae*, and *Mycobacterium* spp. target sequences.

Real-time PCR reactions were performed in a final volume of 20 μL using *Aeromonas veronii* qPCR test kit (YouSeq Ltd., Winchester, UK), *Photobacterium damselae* qPCR test kit (YouSeq Ltd., UK) and *Mycobacterium* spp. qPCR test kit (YouSeq Ltd., UK) in accordance with the manufacturer’s protocol. All assays were performed at the Aria Mx thermal cycler (Agilent Technologies, Santa Clara, CA, USA) under the following conditions: denaturation at 95 °C for 3 min, followed by 45 cycles of annealing/extension at 95 °C for 15 s and 60 °C for 1 min.

### 2.8. Whole-Genome Sequencing and Data Analysis

The *Aeromonas veronii* strains Thesp1 and Aetol1 were subjected to whole-genome sequencing, using Illumina’s Novaseq platform producing 150 bp paired-end reads. The NCBI Prokaryotic Genome Annotation Pipeline (PGAP) tool was used to identify the taxonomy of the assembled genomes by computing the Average Nucleotide Identity (ANI) [25]. The sequencing products were quality checked and assembled using fastp v0.23.4 [26] and skesa v2.3.0 [27]. The assembled contigs were annotated using Prokka v1.14.6 [28,29] and the sequence type (ST) was identified using the PubMLST platform [30]. FastANI was used to define the pairwise genome similarity [31] and phylogenetic analysis was implemented against representative and *Aeromonas veronii* strains from Aegean Sea [32] using Clustal Omega (version 1.2.4) [33] on the concatenated sequences of the six-gene MLST scheme for *Aeromonas* spp. including the gltA, groL, gyrB, metG, ppsA, and redA genes.

Table 1 presents the number of samples per analysis mentioned in the Materials and Methods section.

## 3. Results

### 3.1. Gross Pathology and Microscopic Examination

**Figure 2:**
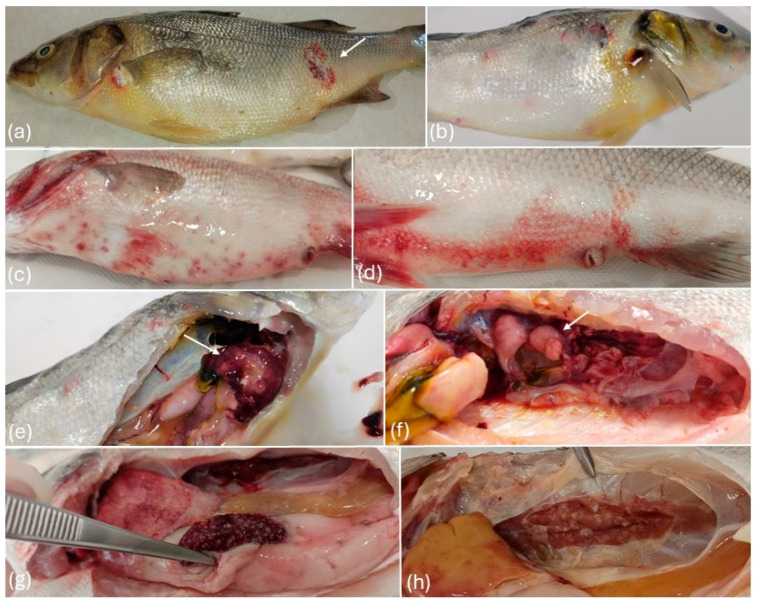
(**a**) Icteric appearance and ulcer (arrow) on the skin, (**b**) icteric appearance of the skin, (**c**) petechiae and ulcers in the ventral skin, (**d**) ecchymosis in the skin and distension of the cloaca, (**e**) abscesses (arrow) and nodules in the liver, (**f**) renal abscesses (arrow), (**g**) whitish nodules and abscesses in the spleen, (**h**) renal nodules.

Pale or yellow coloration of the skin was observed in most of the affected individuals, frequently accompanied by petechiae or ecchymoses on the skin, fins, and cloaca (Figure 2). Additionally, erosions and ulcers were frequently noted both on the ventral and dorsal skin, as well as on the maxilla and opercula. The gills appeared pale, often with hemorrhages and excessive mucus production. Other signs occasionally observed were coelomic distension due to ascites, and ocular lesions, including exophthalmos and corneal ulcers. 

Internally, diffuse congestion and widespread petechiae or hemorrhages were observed in acute courses of the disease. Splenomegaly and renomegaly were consistently present in all affected individuals accompanied in most of the cases, by multifocal to coalescing areas of necrosis and abscess formation in the spleen, liver, and kidney. As the disease progressed, these organs developed irregular, whitish to pale nodules of varying sizes, both on the surfaces and within the parenchyma. In severe cases, similar nodules were also present throughout the liver, heart, gonads, and other visceral organs.

Discoloration or yellow coloration of the liver was observed in the majority of the individuals. The digestive tract was also distended in most of the fish examined, with yellow fluid or caseous material present in the intestinal lumen. Ascites was also one of the main findings during necropsy. 

Parasitological examination revealed gill infections by the copepods *Lernanthropus kroyeri* and *Caligus minimus* and the monogenean *Diplectanum aequans*, with parasite loads consistent with those typically observed in fish of this age group. Additionally, low numbers of the myxosporean *Sphaerospora dicentrarchi* were identified in intestinal smears.

Ziehl–Neelsen-stained impression smears of the kidney and spleen showed no evidence of acid-fast bacteria.

### 3.2. Histopathology

All examined sections of the liver, kidney, and spleen showed the presence of histopathological lesions. In general, microscopic observation showed the development of granulomas along with hemorrhagic and necrotic areas infiltrated by inflammatory cells. The infiltrate was mixed lymphohistiocytic with multinucleated giant cells and many scattered neutrophils.

**Figure 3:**
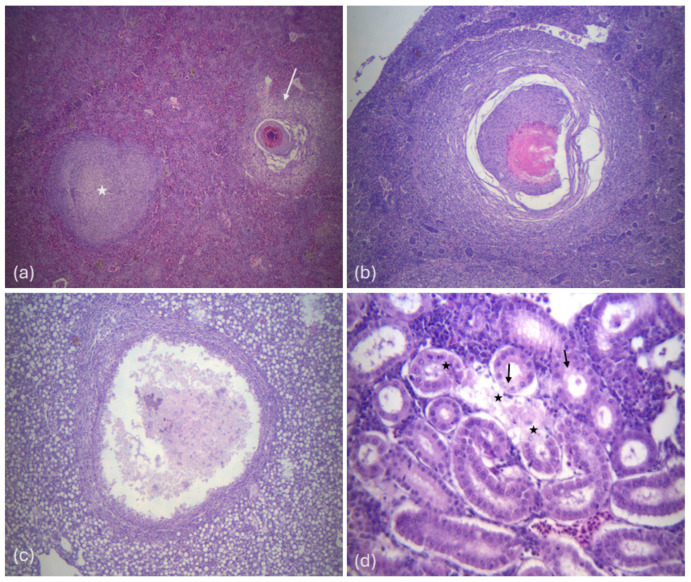
(**a**) Histological section of the spleen; displays an early-stage granuloma (star) as well as a fully developed granuloma (arrow) (magnification ×40), (**b**) spleen tissue section showing a typical granuloma (magnification ×100), (**c**) liver histological section showing an abscess formation. There is a visible central region of amorphous necrotic proteinaceous material within a collagenous capsule. (magnification ×100), (**d**) kidney histological section showing tubules with necrosis (star), and degeneration of some epithelial cells (arrows) (magnification ×400).

Large focal or multifocal granulomas were observed throughout the parenchyma of the organs examined, characterized by extensive, well-defined necrotic areas containing tissue debris and bacteria, at the center, surrounded by multiple layers of inflammatory cells and loose connective tissue (Figure 3).

Additional histopathological findings included abscess formation in the liver and spleen, tubular degeneration or necrosis accompanied by hemorrhages in the kidney and hemorrhages in the spleen.

### 3.3. Phenotypic Characteristics

**Figure 4:**
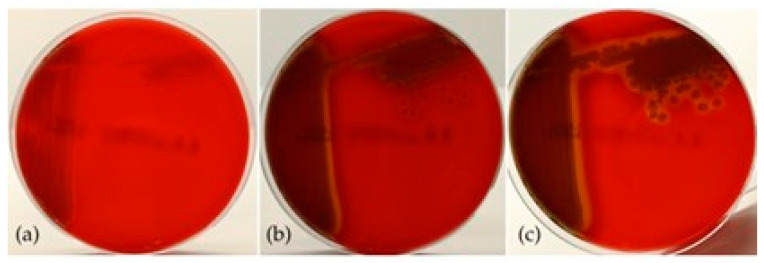
Hemolytic activity in BA, after (**a**) 24 h, (**b**) 48 h, and (**c**) 72 h of incubation.

**Figure 5:**
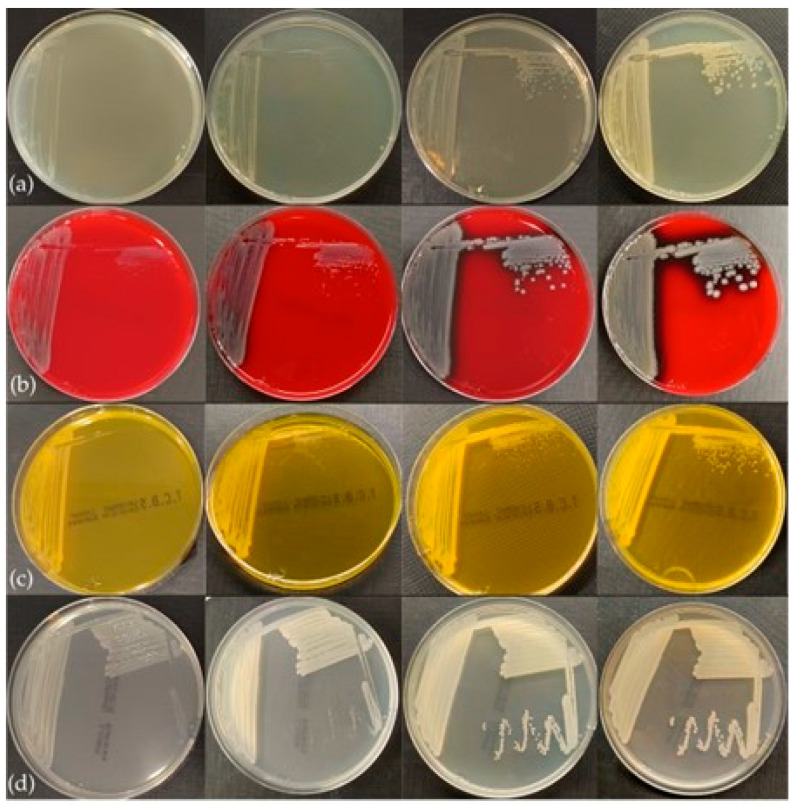
Colony growth in 18, 24, 48, and 72 h of incubation in four media: (**a**) TSA, (**b**) BA, (**c**) TCBS, (**d**) MH.

The bacteria grew well on all nutrient substrates following 18–24 h of incubation. The colonies exhibited phenotypic similarities characterized by a smooth, circular morphology and an opaque appearance. The coloration of the colonies varied depending on the cultivation medium and the duration of incubation. On Blood Agar (BA), colonies initially appeared off-white, transitioning to greyish after 48 h, and demonstrated hemolytic activity after 24 h of incubation (Figure 4). On Tryptic Soy Agar (TSA) and Mueller–Hinton Agar (MH), colonies exhibited a whitish coloration at 24 h, evolving to a beige coloration after 48 h, with no production of pigmentation noted. Additionally, yellowish coloration of both the colonies and the substrate was observed on Thiosulfate Citrate Bile Salts (TCBS) agar after 18 h of incubation (Figure 5).

Microscopic evaluation of the colonies revealed the presence of rod-shaped, motile bacilli, measuring from 1.2 μm to 2.5 μm in length. Gram staining further demonstrated that these bacteria are Gram-negative (Figure S1 in the Supplementary Materials).

Antimicrobial susceptibility testing conducted on various bacterial strains isolated from the regions of Thesprotia and Aetoloakarnania demonstrated sensitivity of the bacteria to all antibiotics evaluated in this study.

All the phenotypic characteristics of *Aeromonas veronii* are presented in Table 2.

### 3.4. Biochemical Profiles

All isolates tested were oxidase- and catalase-positive and displayed positive results in the motility test.

According to the results of the GNA+B-ID System software (version 1.2), the strains were identified as *Aeromonas veronii* biovar *sobria*, while they were identified as *Aeromonas sobria* by the VITEK^®^ 2 software (version 9.01), respectively.

Further details of the comprehensive profiles of each system are presented in Table S1 in the Supplementary Materials.

### 3.5. Molecular Characterization

Real-time PCR analysis confirmed the presence of *Aeromonas veronii* DNA in all tested colony extracts. Additionally, all spleen tissue samples were tested positive for *A. veronii*, while no amplification was observed for *Mycobacterium* spp. or *Photobacterium damselae*.

### 3.6. Genomic and Phylogenetic Characterization

**Figure 6:**
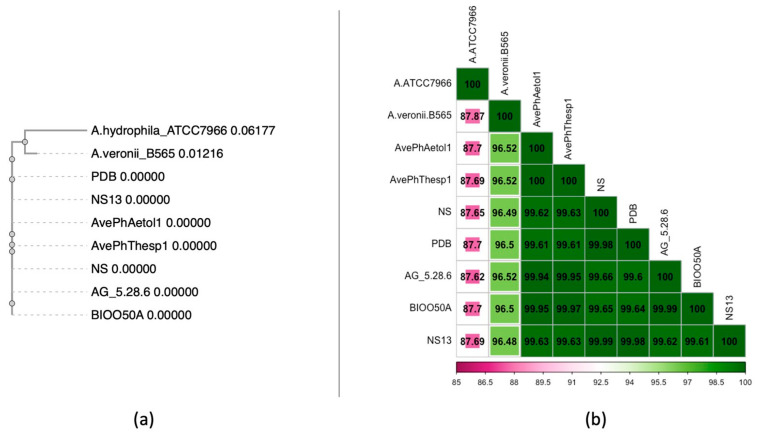
(**a**) Phylogenetic tree of the assembled genomes, closely related and representative isolates using the MLST gene sequences, (**b**) Heatmap of the pairwise genome comparisons using the whole-genome assemblies.

Taxonomic classification of the sequencing products validated the isolation of *Aeromonas veronii* (taxid 654) samples. Following quality assessment of the 150 bp paired-end reads, the resulting ~12.5 Mbp sequencing products of each isolate were assembled into 113 (N50: 86,929) and 112 contigs of the AveP hThesp1 and AvePhAetol1 samples, respectively, with N50 value of 86.9 kb for both assembled genomes. Both isolates are assigned to ST23. Comparative analysis against *Aeromonas veronii* strains isolated from both Ionian (AvePhThesp1, AvePhAetol1) and Aegean Sea (NS13, NS, AG_5.28.6, PDB, BIOO50A) verified the expected 100% identity of the MLST genes and distinct genetic composition for representative isolates (*A. hydrophila* ATCC7966 and *A. veronii* B565) as shown in the phylogenetic tree (Figure 6a). Further analysis of the whole-genome average nucleotide identity revealed that on genome-wide level the Ionian Sea genomes were 100% identical, while the similarity level against the Aegean Sea genomes was slightly lower ranging between 99.61% and 99.97% (Figure 6b). As expected, more pronounced genetic variation is observed in the representative genome assemblies 96.48–96.52% for *A. veronii* B565 and 87.62–87.87% for *A. hydrophila* ATCC7966.

## 4. Discussion

A severe outbreak of aeromoniasis in farmed European seabass on the Greek coast of the Ionian Sea is described in this paper, composing the first documented record of the disease in this region. The etiological agent identified was *Aeromonas veronii*, a Gram-negative bacterium associated with significant losses in European seabass in the Aegean and the Black Sea [17,18,33], as well as other fish species of both freshwater and marine environments [34,35]. The outbreak, which occurred during the summer and autumn of 2024, was characterized by high morbidity and considerable cumulative mortality, resulting in important biomass losses and significant economic losses for local aquaculture producers.

The affected fish exhibited signs such as anorexia, lethargic behavior, and sudden mortalities. Severe macroscopic lesions were also observed, including petechiae and extensive hemorrhages on the external surfaces and internal organs. Necrotic foci, abscesses, and granulomatous inflammation were also documented in internal organs such as spleen, kidneys, and liver. In cases exhibiting more severe manifestations, an icteric appearance was observed in the skin, gills, and coelomic cavity organs. Furthermore, progressive erosion and ulceration of the skin, extending into the underlying tissues, were recorded.

These findings align with those documented in previous reports of *Aeromonas veronii* infection in farmed seabass across other Mediterranean regions [17,18,34,36], where anorexia, icteric appearance, ulcerative skin lesions and extensive internal organs lesions were observed in commercial size fish, correlated with rising water temperatures. Further examination of *Aeromonas veronii* outbreaks within other fish species revealed characteristic ulcerative lesions and hemorrhages in both external and internal tissues. Such pathology has been reported in channel catfish in Vietnam [6], freshwater sleepers in China [9], *Cyprinus carpio* in Korea [37], and snakehead fish in China [38]. Notably, *Aeromonas veronii* has also been linked to outbreaks among smaller fish sizes, including juveniles of *Myxocyprinus asiaticus* in China, which exhibited symptoms such as fin rot, swollen cloacas, pale gills, and enlarged hematopoietic tissues [8]. Recent reports have indicated that lesions associated with acute septicemia in *Lateolabrax maculatus* have been rendered to *Aeromonas veronii* infections in China [39].

In the Mediterranean, motile aeromonad diseases caused by various *Aeromonas* species have been associated with hemorrhages and splenomegaly in seabass (150 and 330 g) and *Pagellus puntazzo* (45 g) in Greece [40], as well as in seabass (weighing 100 g) farmed along the Black Sea coasts of Turkey [18]. In these outbreaks, the causative agents were identified as *A. hydrophila* and *A. salmonicida* subsp*. achromogenes*, respectively. In smaller fish sizes, a 3.8% cumulative mortality was reported among juvenile seabass (9 g) in Spain due to *A. salmonicida* subsp*. salmonicida*, accompanied by symptoms including ulcerative lesions in the skin and muscle tissue as well as splenic enlargement [41]. A similar outbreak in seabream in Gran Canaria (Atlantic) was also reported, where *A. salmonicida* subsp. *salmonicida* led to hyperacute disease, resulting in mortality rates of 6–7% within the first three days [42].

The contribution of various pathogens, including gill parasites such as *Lernanthropus kroyeri*, *Diplectanum aequans*, and *Caligus minimus*, appeared to be of minor significance in relation to the observed mortality. The parasitic loads detected were within the typical range for seabass exceeding 250 g within this geographical region. Furthermore, other potential pathogens capable of inducing granulomatous inflammation, such as species of *Mycobacterium*, were included in the differential diagnosis and excluded. These findings suggest that, while these pathogens are present or can potentially be detected, they are unlikely to be significant contributors to morbidity and mortality during that outbreak.

The histopathological findings observed in this study are consistent with previous reports documenting similar pathological conditions in *A. veronii*-infected individuals. Specifically, Smyrli et al. (2017) [17] observed multifocal liquefactive necrosis in the spleen and abscesses in the liver of diseased seabass in the Aegean Sea. In the study of Karatas et al. (2023) [43], vacuolation and necrosis in parenchyma cells were observed, along with hemorrhages in the liver of European seabass in Turkey. Similarly, Liu et al. observed swelling and hemorrhages in the liver blood sinuses, edema in renal tubules, and extensive necrosis and vacuolation of the spleen in bronze gudgeon (*Coreius heterodon*). A study by Bispo dos Santos et al. noted diffuse vacuolar depletion and focal mononuclear inflammatory infiltrates in the liver of Nile tilapia, as well as hyperplasia of melano-macrophage centers and focal interstitial leukocyte infiltrate in the spleen. In *Cyprinus carpio* from Korea, Jin Ha Yu et al. documented numerous bacterial invasions in the hemorrhagic splenic pulp, along with multiple hemorrhagic foci in the hematopoietic tissue, and necrotized renal tubules and glomerular destructions. Additionally, necrotic areas in the spleen and renal tissue, particularly in the renal tubules, were observed in Channel catfish in China. Summarizing all these reports, it is evident that *A. venonii* induces a severe systemic response, characterized by extensive tissue damage and distinct histopathological changes that are consistent across various fish species.

Aeromonads grow very fast in typical nutrient media, forming circular, convex colonies, measuring approximately 2–3 mm in diameter following incubation for 24 to 48 h [20,32]. Aeromonads are further categorized based on their motility and the ability to produce brown pigmentation [20,32,44,45,46,47].

In our study, all isolates exhibited motility, grew on all the nutrient substrates examined—even in TCBS—after 18 h of incubation, and did not produce any brown pigmentation. This specific set of characteristics reveals phenotypic variations of the Ionian Sea isolates in comparison to the Aegean Sea ones, not allowing their classification into any of the three groups previously suggested by Smyrli et al. [32].

Regarding commercial biochemical identification systems, such as API20E and BIOLOG GENIII Microplate, there are limitations in accurately identifying fish pathogens and particularly aeromonads, at species level [32]. Similarly, we have observed such restrictions with the biochemical testing systems used in this study (Microgen GnA+B-ID and VITEK 2), especially regarding the identification of the Vibrionaceae family members at the species level (personal data). In this research, the isolates were biochemically analysed using Microgen GnA+B-ID and characterized as *A. veronii* bv. *sobria*, while when examined with VITEK 2, they were identified as *A. sobria*. Our findings aligned with general features of most species within the *Aeromonas* genus, as all isolates demonstrated typical biochemical properties that have been previously described by Abbott et al. (2003) [44] and Janda et al. (2010) [46], including positive oxidase and catalase reactions, positive β-galactosidase activity, mannitol fermentation, lack of urea hydrolysis, negative malonate, and negative adonitol reactions. In relation to L-arabinose, a key parameter for differentiating *Aeromonas sobria* and *Aeromonas veronii* species complex from other *Aeromonas* species [44], all our isolates were negative to L-arabinose and thus characterized as *Aeromonas veronii*. Moreover, ornithine decarboxylase has been recognized as a significant feature for classifying *Aeromonas veronii* within the biovars *veronii* and *sobria* [44]. Our isolates were tested negative, and this property is placing Ionian isolates in the sobria biovar.

Regarding the differences we noticed in our isolates compared to other *Aeromonas* species, one isolate in our study was tested positive for inositol, while *Aeromonas* species are tested negative for inositol [44]. Additionally, while *Aeromonas veronii* isolates generally exhibit positive lysine decarboxylase activity [44], two isolates from this study were found to be negative. We also observed variability in sucrose fermentation, with two of the four Ionian isolates testing negative, in opposition to the typical positive responses seen in most *Aeromonas* species [44,46], as well as the consistently positive results from isolates reported from the Aegean and Black Seas [17,32,34,36].

Regarding the halose fermentation, three Ionian isolates exhibited negative results, in contrast with the majority of *A. veronii* isolates, which gave positive results [9,10,32,48,49]. A comparative analysis including additional isolates could provide more definitive insights and aid in the development of standardized biochemical profiles for *A. veronii* species.

Biofilm formation is a virulence factor related to the pathogenicity of Aeromonas and strongly influences the occurrence of diseases in fish and humans [50,51]. In the present study, all Aeromonas isolates were classified as non-biofilm producers in terms of their ability to form biofilms. This finding is consistent with previous studies that have reported similar results [51,52].

Understanding the population structure of the bacterium is crucial for the development of effective strategies for the treatment of infections. To gain insight into the genetic relatedness of *A. veronii* isolated from different areas, WGS analysis was performed. The whole genome sequence analysis confirmed the successful isolation of *Aeromonas veronii* strains. Both AvePhThesp1 and AvePhAetol1 are classified in the same sequence type (ST23). Comparative genomic analysis revealed the 100% identity of MLST genes between the two strains and significant genetic similarity with *A. veronii* strains from the Aegean Sea reported by Smyrli et al. (2019) [32]. Lower genetic similarity level was observed with the reference strain *A. veronii* B565, while clear genetic divergence was noted when compared to *A. hydrophila*. Additionally, the analysis of the genome-wide average nucleotide identity revealed highly identical sequences between AvePhThesp1 and AvePhAetol1, and slightly reduced similarity (99.61% to 99.97%) with *Aeromonas veronii* strains from the Aegean Sea. Despite the close genetic relationships among *A. veronii* isolates derived from different geographical areas in Greece, phenotypic differences such as motility and pigmentation production, have been observed. To enhance our understanding of these phenotypic variations and the overall characteristics of *A. veronii*, further systematic investigation into specific genomic factors is necessary. This knowledge will be crucial for the further development of effective vaccines.

## 5. Conclusions

This study presents the first outbreak of *Aeromonas veronii* infection in farmed European seabass along the Ionian Sea coast of Greece. The outbreak, marked by high morbidity and mortality, caused substantial economic losses and displayed clinical and pathological features consistent with those reported in previous *A. veronii* infections in other regions. Whole-genome sequencing and phylogenetic analysis confirmed high genetic homogeneity among the Ionian strains and with *A. veronii* strains from the Aegean Sea, reinforcing their classification within sequence type ST23. Despite genetic similarities, notable phenotypic and biochemical differences—particularly in motility, pigmentation, and metabolic profiles—were observed, indicating regional phenotypic variability. These findings underscore the need for continued surveillance, detailed genomic and phenotypic characterization, and the development of targeted prevention strategies, including autogenous vaccines, to mitigate the impact of *Aeromonas veronii* in Mediterranean aquaculture systems.

## Figures and Tables

**Table 1:** Number of samples per analysis.

Region	Fish Farms (Identification Number)	Necropsy ^1^	Histology ^1^	Cultures ^1^	Antimicrobial Susceptibility Tests ^2^	Biochemical Identification ^2^	Biofilm Formation Ability ^2^	Real-Time PCR (Colonies)	Real-Time PCR (Pooled Spleens)	Whole Genome Sequencing ^2^
Thesprotia	#1	94	3	94	15	1	1	4	7	1
	#2	80	3	80	13	1	1	3	3	-
	#3	66	3	66	11	1	1	3	2	-
	#4	78	4	78	13	-	1	3	3	-
	#5	68	3	68	11	-	1	2	2	-
	#6	82	3	82	12	-	1	2	2	-
Aetoloakarnania	#1	56	3	56	9	1	1	4	4	1
Total	7	524	22	524	84	4	7	21	23	2

Legend: ^1^ the number of individuals tested, ^2^ the number of isolations tested.

**Table 2:** Phenotypic characteristics of the *Aeromonas* veronii isolates from Greece.

Region	Isolates	Antimicrobial Resistance Profiles ^a^						Biofilm ^b,c^			Motility	Pigmentation ^c^
		OA	DO	OT	SXT	FFC	UB	ODc	OD	Formation Ability ^c^		
Ionian Sea	Thesp1	S	I	S	R	S	S	0.052	0.038	NB	+	−
	Thesp2	I	I	I	I	S	S	0.052	0.041	NB	+	−
	Thesp3	I	I	I	I	S	S	0.052	0.045	NB	+	−
	Thesp4	I	I	I	I	S	S	0.052	0.040	NB	+	−
	Thesp5	I	I	I	I	S	S	0.052	0.040	NB	+	−
	Thesp6	I	I	I	I	S	S	0.052	0.035	NB	+	−
	Aetol1	S	S	S	S	S	S	0.052	0.043	NB	+	−
East Aegean Sea	AG 5.28.6	S	S	S	S	S	S	ND	ND	ND	+	ND
	BIOO50A	S	S	S	S	S	S	ND	ND	ND	+	ND
West Aegean Sea	NS13	S	S	S	S	S	S	ND	ND	ND	−	−
	PDB	S	S	S	S	S	S	ND	ND	ND	+	+
	NS	S	S	S	S	S	S	ND	ND	ND	−	−

Legend: (a) Antimicrobial acronyms: OA, oxolinic acid; DO, doxycycline; OT, oxytetracycline; SXT, sulfamethoxazole/trimethoprim; FFC, florfenicol; UB, flumequine; (b) ability to form biofilm according to Borges et al. [24]: ODc, three standard deviations above the average optical density of the negative control; OD, mean optical density of the isolate; NB, no biofilm producer (OD < ODc); W, weak biofilm producer (ODc < OD ≤ 2 × ODc); M, moderate biofilm producer (2 × ODc < OD ≤ 4 × ODC); S, strong biofilm producer (4 × ODc < OD), (c) ND, not defined.

## Data Availability

This Whole Genome Shotgun project has been deposited at DDBJ/ENA/GenBank under the BioProject accession PRJNA1224816. The version described in this paper includes the analysis of the sample under the accession JBLUPC000000000.1 (AvePhThesp1) and JBLUPD000000000.1 (AvePhAetol1).

## Supplementary Materials

The following supporting information can be downloaded at: https://www.mdpi.com/article/10.3390/pathogens14060587/s1, Figure S1: Gram-negative stained Aeromonas veronii bacteria; Table S1. Biochemical test results with the GNA+B-ID System and VITEK^®^ 2 Automated System.
