# Supplementary material for: First Outbreak of Aeromoniasis, Caused by Aeromonas veronii, in Farmed European Seabass (Dicentrarchus labrax) in the Ionian Sea, Greece
Source: Pathogens. 2025 Jun 14;14(6):587. doi: 10.3390/pathogens14060587 (PMC12195627; doi:10.3390/pathogens14060587)
Supplement: Supplementary file 1 [file pathogens-14-00587-s001.zip › Table S1.pdf]

Table S1. Biochemical test results with the GNA+B-ID System and VITEK® 2 Automated System.

Biochemical Parameter	Thesp1	Thesp2	Thesp3	Aetol1
GNA+B-ID System				
β- galactosidase (ONPG)	+	+	+	+
Lysine decarboxylase (LYS)	+	-	-	+
Ornithine decarboxylase (ORN)	-	-	-	-
Citrate utilization (CIT)	-	-	-	-
Nitrate reduction (NIT)	+	+	+	+
Motility (MOT)	+	+	+	+
H2S production (H2S)	-	-	-	-
Urease (UR)	-	-	-	-
Tryptophan deaminase (TDA)	-	-	-	-
Indole production (IND	-	-	-	-
Acetoin production (VP)	+	+	+	-
Gelatinase (GEL)	+	+	+	+
Oxidase (OXI)	+	+	+	+
Glucose (GLU)	+	+	+	+
Malonate (MAL)	-	-	-	-
Mannitol (MAN)	+	+	+	+
Xylose (XYL)	-	-	-	-
Inositol (INO)	-	-	-	+
Sorbitol (SOR)	-	-	-	-
Rhamnose (RHA)	-	-	-	-
Sucrose (SUC)	+	-	-	+
Lactose (LAC)	-	-	-	-
Arabinose (ARA)	-	-	-	-
Adonitol (ADO)	-	-	-	-
Raffinose (RAF)	-	-	-	-
Salicin (SAL)	-	-	-	-
Arginine (ARG)	+	+	+	+
VITEK® 2 Automated System				
Ala-Phe-Pro- Arylamidase (APPA)	-	-	-	-
Adonitol (ADO)	-	-	-	-
L-Pyrrolydonyl- Arilamidase (PyrA)	-	-	-	-
L-Arabitol (IARL)	-	-	-	-
D- Cellobiose (dCEL)	-	-	-	-
Beta- Galactosidase (BGAL)	+	+	+	+
H2S Production (H2S)	-	-	-	-
Beta-N- Acetyl- Glucosaminidase (BNAG)	+	+	+	+
GlutamylArylamidase pNA (AGLTp)	+	+	-	-
D-Glucose (dGLU)	+	+	+	+
Gamma-Glutamyl- Transferase (GGT)	+	+	-	+
Fermentation Glucose (OFF)	+	+	+	+
Beta-Glucosidase (BGLU)	-	-	-	-
D-Maltose (Dmal)	+	+	+	+
D-Mannitol (Dman)	+	+	+	+
D-Mannose (Dmne)	+	+	+	+
Beta- Xylosidase (Bxyl)	-	-	-	-

Beta- Alaninearylamidase pNA (BaLAP)	-	-	-	-
L-Proline Arylamidase (ProA)	+	+	+	+
Lipase (LIP)	-	-	-	-
Palatinose (PLE)	-	-	-	-
Tyrosine Arylamidase (TyrA)	+	+	+	+
Urease (URE)	-	-	-	-
D-Sorbitol (dSOR)	-	-	-	-
Saccharose/Sucralose (SAC)	+	-	-	+
D-Tagatose (Dtag)	-	-	-	-
D-Trehalose (Dtre)	-	-	+	-
Citrate (Sodium) (CIT)	-	-	-	-
Malonate (MNT)	-	-	-	-
5-Keto-D-Gluconate (5KG)	-	-	-	-
L-Lactate Alkalinisation (ILATk)	-	-	-	-
Alpha-Glucosidase (AGLU)	-	-	-	-
Succinate Alkalinisation (SUCT)	+	+	+	+
Beta-N-Acetyl-Galactosaminidase(NAGA)	+	+	+	+
Alpha-Galactosidase (AGAL)	-	-	-	-
Phosphatase (PHOS)	-	-	-	-
Glycine Arylamidase (GlyA)	-	-	-	-
Ornithine Decarboxylase (ODC)	-	-	-	-
Lysine Decarboxylase (LDC)	-	-	-	+
L-Histidine assimilation (IHISa)	-	-	-	-
Coumarate (CMT)	+	+	+	+
Beta-Glucoronidase (BGUR)	-	-	-	-
O/129 Resistance (O129R)	+	+	+	+
Glu-Gly-Arg-Arylamidase (GGAA)	+	+	-	+
L-Malate assimilation (IMLTa)	-	-	-	-
Ellman (ELLM)	+	+	+	+
L-Lactate assimilation (ILATa)	-	-	-	-

Symbols: (+) positive reaction, (-) negative reaction.
