# Supplementary figures and images for: First Outbreak of Aeromoniasis, Caused by Aeromonas veronii, in Farmed European Seabass (Dicentrarchus labrax) in the Ionian Sea, Greece
Source: Pathogens. 2025 Jun 14;14(6):587. doi: 10.3390/pathogens14060587 (PMC12195627; doi:10.3390/pathogens14060587)

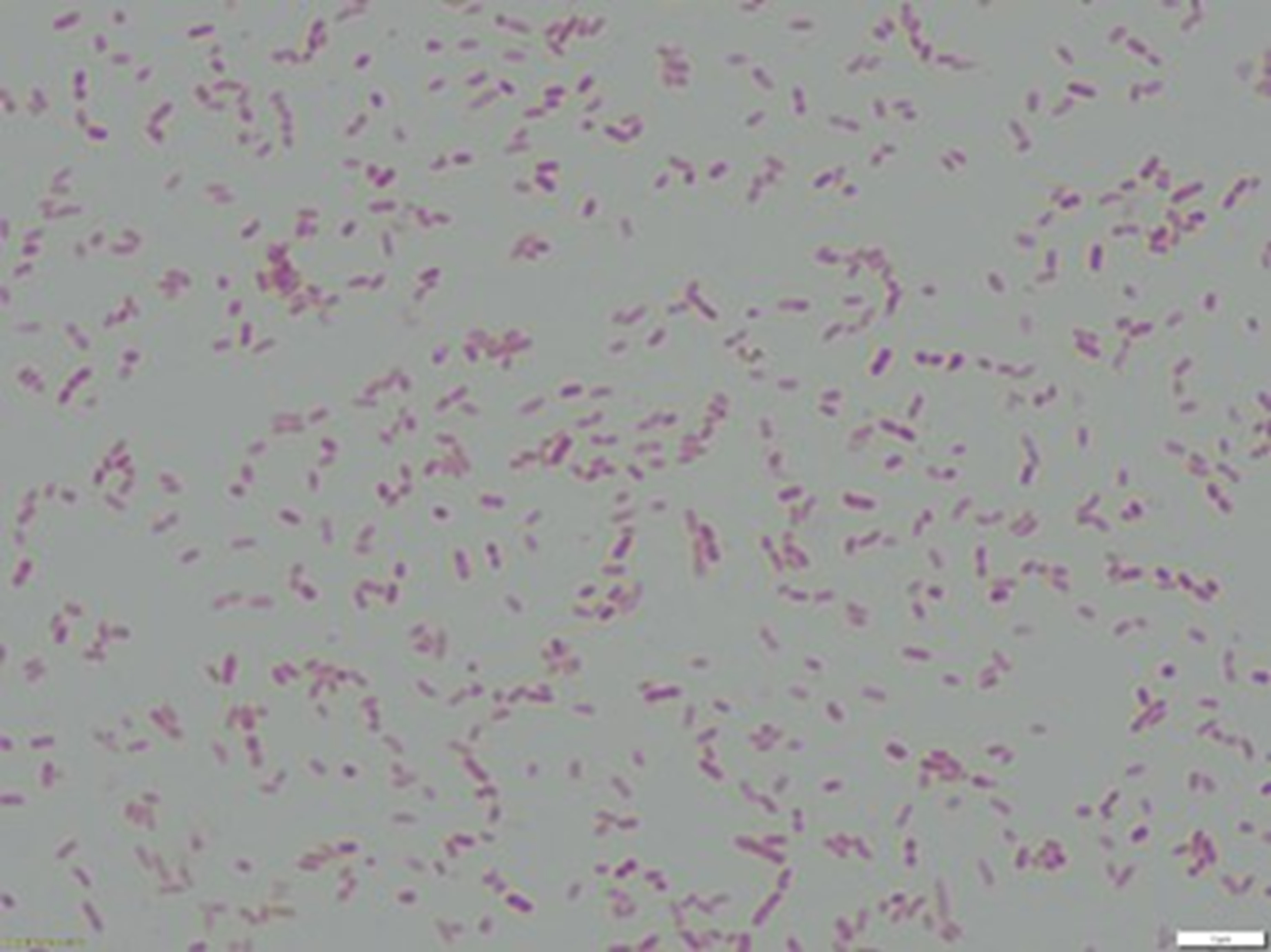

Supplement: Supplementary file 1 [file pathogens-14-00587-s001.zip › Figure S1.jpg]
